# Supplementary material for: Plastid Molecular Chaperone HSP90C Interacts with the SecA1 Subunit of Sec Translocase for Thylakoid Protein Transport
Source: Plants (Basel). 2024 May 1;13(9):1265. doi: 10.3390/plants13091265 (PMC11085213; doi:10.3390/plants13091265)
Supplement: Supplementary file 1 [file plants-13-01265-s001.zip › plants-2951497-supplementary.pdf]

Supplementary Materials:

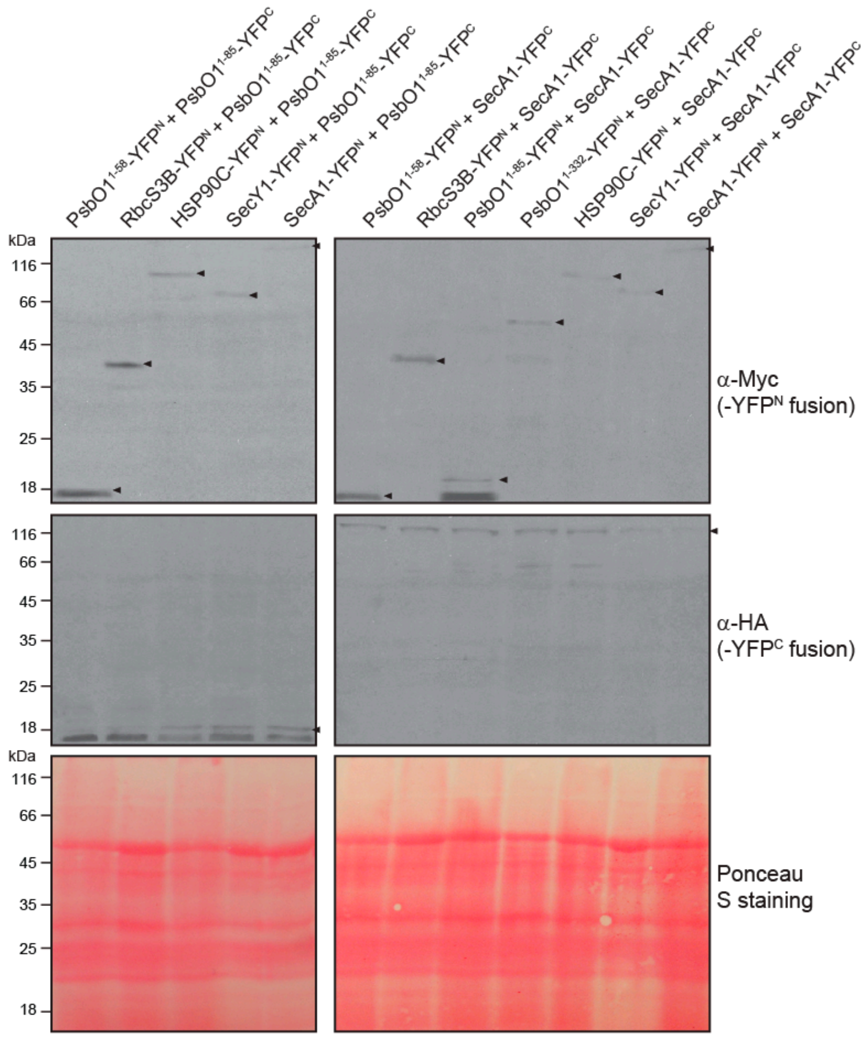

Figure S1: Immunoblot of transiently expressed soluble half-YFP fusion proteins in tobacco leaf. Protein bands of interest are marked by the arrows, and anti-Myc and anti-HA were used to detect YFP N- and C-terminal half-fusion proteins, respectively.

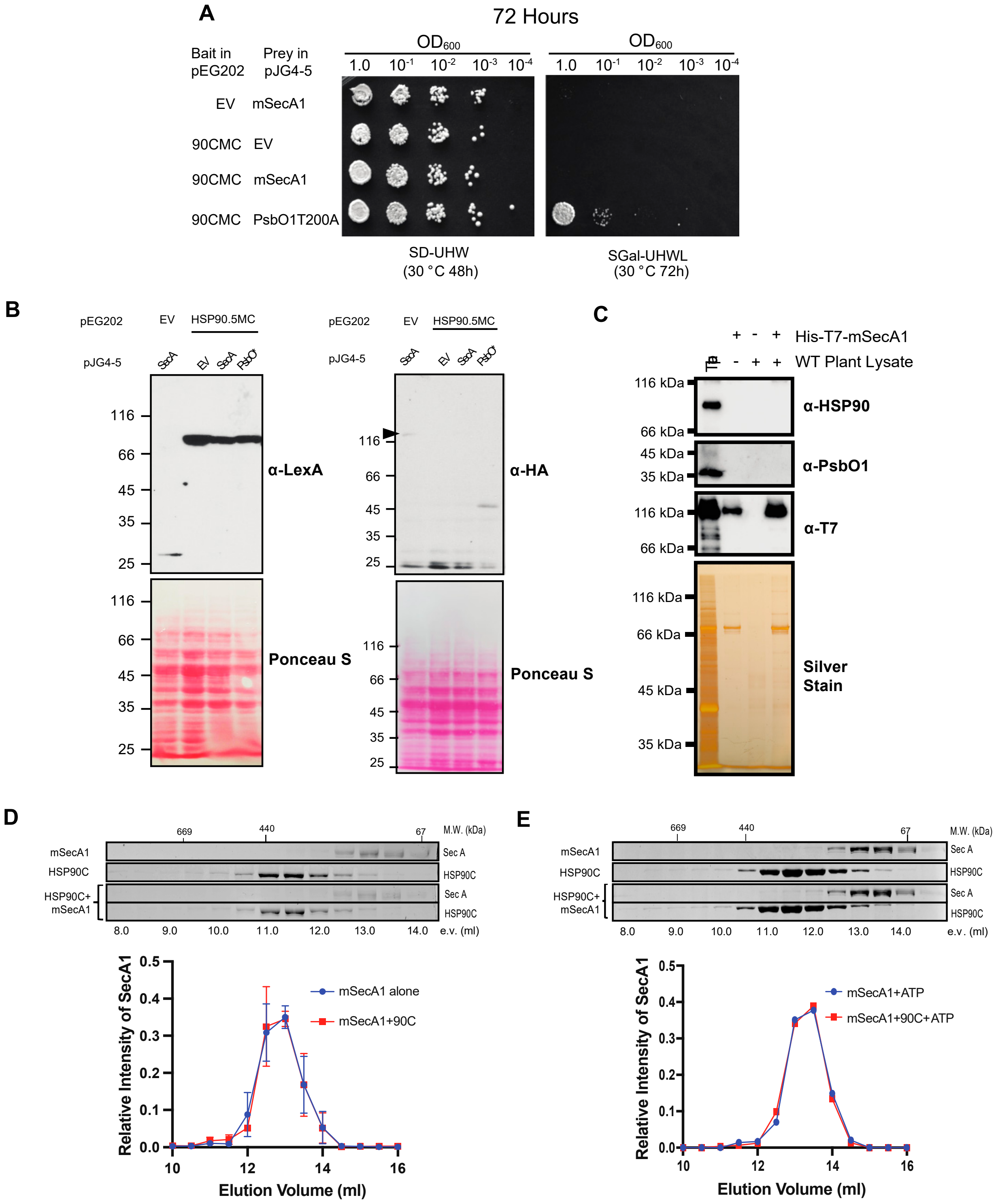

Figure S2: Lack of significant direct interaction between HSP90C and SecA1 explored through in vitro and heterologous expression assays. (**A**) Yeast-two-hybrid assay between SecA1 and the HSP90C middle and C-terminal domain. (**B**) Immunoblot confirmation of the presence of bait and prey proteins from the yeast two-hybrid assay using anti-LexA and anti-HA antibodies. (**C**) Immunoblot analysis of the pull-down assay using immobilized mSecA1 protein and wild-type plant lysate prey. (**D**) Assessment of the elution profiles obtained from size exclusion chromatography analysis using 100 µg of purified mSecA1 and HSP90C. (**E**) Assessment of the elution profiles obtained from size exclusion chromatography analysis using 100 µg of purified mSecA1 and HSP90C when pre-treated with 5 mM ATP.

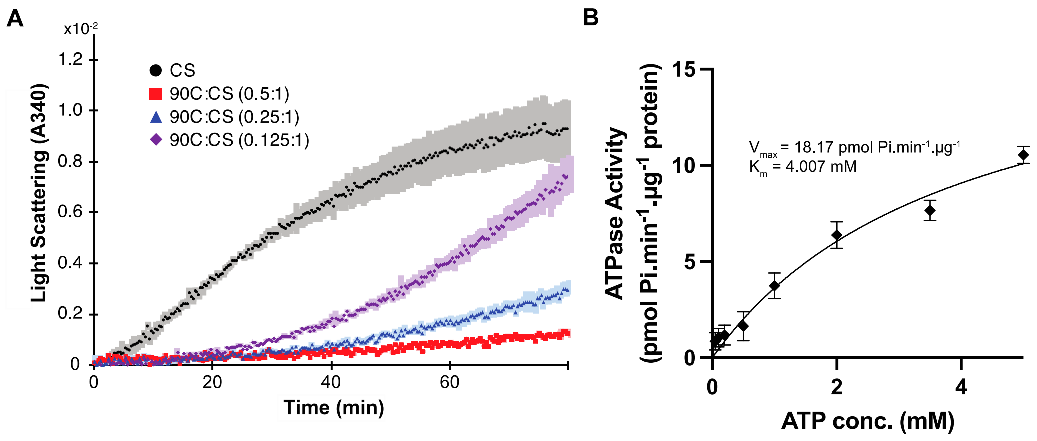

Figure S3: Verification of HSP90C's chaperone activity. (**A**) Citrate synthase (CS, 1 μM) was incubated in the presence or absence of different amounts of HSP90C (90C). The molar ratios of CS:HSP90C are indicated in parentheses. The shaded areas represent the SEM from three trials for each molar ratio of the chaperone used. (**B**) Kinetics assay of HSP90C in hydrolyzing ATP. The specific activity was measured as Radicicol-sensitive activity and error bars represent SD from six experimental repeats for each ATP concentration.
